# Effect of PACAP on Hypoxia-Induced Angiogenesis and Epithelial–Mesenchymal Transition in Glioblastoma

**DOI:** 10.3390/biomedicines9080965

**Published:** 2021-08-05

**Authors:** Grazia Maugeri, Agata Grazia D’Amico, Salvatore Saccone, Concetta Federico, Daniela Maria Rasà, Rosario Caltabiano, Giuseppe Broggi, Salvatore Giunta, Giuseppe Musumeci, Velia D’Agata

**Affiliations:** 1 Department of Biomedical and Biotechnological Sciences, Section of Anatomy, Histology and Movement Sciences, University of Catania, 95100 Catania, Italy; graziamaugeri@unict.it (G.M.); rasa.daniela@gmail.com (D.M.R.); sgiunta@unict.it (S.G.); g.musumeci@unict.it (G.M.); 2 Department of Drug Sciences, University of Catania, 95100 Catania, Italy; agata.damico@unict.it; 3 Department of Biological, Geological and Environmental Sciences, Section of Animal Biology, University of Catania, 95123 Catania, Italy; saccosal@unict.it (S.S.); federico@unict.it (C.F.); 4 Department of Neuroscience Rita Levi Montalcini, Neuroscience Institute Cavalieri Ottolenghi, University of Turin, 10124 Turin, Italy; 5 Department of Medical and Surgical Sciences and Advanced Technologies “G.F. Ingrassia”, Anatomic Pathology, University of Catania, 95123 Catania, Italy; rosario.caltabiano@unict.it (R.C.); giuseppe.broggi@gmail.com (G.B.)

**Keywords:** PACAP, glioblastoma, hypoxia, angiogenesis, VEGF, epithelial–mesenchymal transition

## Abstract

Pituitary adenylate cyclase-activating polypeptide (PACAP) exerts different effects in various human cancer. In glioblastoma (GBM), PACAP has been shown to interfere with the hypoxic micro-environment through the modulation of hypoxia-inducible factors via PI3K/AKT and MAPK/ERK pathways inhibition. Considering that hypoxic tumor micro-environment is strictly linked to angiogenesis and Epithelial–Mesenchymal transition (EMT), in the present study, we have investigated the ability of PACAP to regulate these events. Results have demonstrated that PACAP and its related receptor, PAC1R, are expressed in hypoxic area of human GBM colocalizing either in epithelial or mesenchymal cells. By using an in vitro model of GBM cells, we have observed that PACAP interferes with hypoxic/angiogenic pathway by reducing vascular-endothelial growth factor (VEGF) release and inhibiting formation of vessel-like structures in H5V endothelial cells cultured with GBM-conditioned medium. Moreover, PACAP treatment decreased the expression of mesenchymal markers such as vimentin, matrix metalloproteinase 2 (MMP-2) and matrix metalloproteinase 9 (MMP-9) as well as CD44 in GBM cells by affecting their invasiveness. In conclusion, our study provides new insights regarding the multimodal role of PACAP in GBM malignancy.

## 1. Introduction

Glioblastoma (GBM) is the most common and aggressive primary brain tumor in adults [1]. The majority of GBMs are classified as primary, since they develop de novo, whereas a small minority, known as secondary GBMs, slowly progress from lower grade of astrocytomas [2]. Among tumors, GBM displays strong proliferation index, diffused infiltration, aberrant angiogenesis and poor outcome [3]. Despite the multimodal therapeutic approach, comprising surgery followed by radiotherapy and treatment with the alkylating agent, Temozolomide, the median survival rate of GBM-affected patients is around 2 years after diagnosis [4]. The highly malignant phenotype of this tumor as well as its resistance to chemotherapy and radiotherapy is exacerbated by the extensive hypoxic areas in the tumor bulk [5]. In these regions, low oxygen tension arises from reduced vascular perfusion or insufficient oxygen diffusion as consequence of higher metabolic demand of the growing tumor [6]. The early response to hypoxia is mediated by hypoxia-inducible factors (HIFs) composed of an α oxygen-related sub-unit (HIFα), including HIF-1α, and an aryl hydrocarbon receptor nuclear translocator (ARNT) sub-unit, also known as HIF-1β, constitutively expressed into the nucleus independently to the presence of oxygen [7]. Under hypoxia, the HIF-1α sub-unit escapes to cytoplasmic degradation by ubiquitin proteasome system, and it translocates into the nucleus, forming a heterodimer with the β-sub-unit. This complex is able to bind hypoxia response elements (HREs), leading to activation of several downstream target genes, including the vascular endothelial growth factor (VEGF), the key player of tumoral angiogenesis [8,9,10,11,12,13,14]. Many papers have confirmed the strategic role carried out by HIF-1α in adaptive response of GBM cells exposed to micro-environmental hypoxia. In fact, HIF-1α not only contributes to aberrant development of new blood vessels in malignant gliomas, but it is also involved in a variety of tumoral biological processes such as cell metabolism, invasion, survival and proliferation [15,16,17]. Furthermore, HIF-1α has been also considered a main factor involved in tumor metastasis by promoting epithelial to mesenchymal transition (EMT) of cancer cells [18,19]. EMT is a crucial biological process whereby cancer cells lose epithelial features toward a more aggressive and invasive mesenchymal phenotype. Accordingly, EMT is involved in malignancy of different solid tumors, including GBM [20]. Recent papers have demonstrated that hypoxic micro-environment within GBM recruits circulating or residential myeloid cells (i.e., macrophages or microglia) into tumoral stroma by triggering the activation of the EMT process involved in tumor malignant progression and invasiveness of cancer cells in surrounding normal tissue brain [21,22].

Pituitary adenylate cyclase-activating polypeptide (PACAP) is a neuropeptide belonging to the (VIP)/secretin/glucagon family. Peptide exists into two active forms of 38 (PACAP38) or 27 amino acid residues (PACAP27), and it exerts its actions by activating three different G protein-coupled receptors: the specific PACAP receptor (PAC1R) and VPAC1 and VPAC2 receptors binding either PACAP or VIP with similar affinity [23]. The stimulation of these receptors triggers different signaling cascades mediated by adenylate-cyclase (AC) or phospholipase-C (PLC) activation as well as calcium-regulated mechanisms [24]. PACAP and its receptors are expressed in several tissues and organs [25,26]. Many papers have described the physiopathological role of this peptide [27,28,29,30,31]. In particular, it plays a protective role against various insults and in different neurodegenerative diseases [32,33,34,35,36,37,38,39]. Intriguingly, the role of PACAP in cancer is controversial and related to the histopathological hallmarks of the tumor [24,40,41,42,43].

The expression of PACAP and its related receptors has been largely demonstrated in human gliomas [44,45,46,47,48]. The database “The Human Protein Atlas” [49] reports a correlation analysis between tumoral *ADCYAP1* mRNA expression and patient clinical outcome. In particular, 71 patients had a low gene expression and less survival probability than another 82 patients showing high *ADCYAP1* expression and high survival probability. However, no significant difference was detected between groups. Many papers have investigated the functional role of the peptide in GBM. In particular, some studies showed the proliferative effect of PACAP on mouse C6 glioma cells [43,50,51] in contrast Vertongen et al. [52], who demonstrated the ability of PACAP to reduce T98G human glioma cells proliferation. In accord, some studies performed on two human GBM cell lines demonstrated that PACAP agonists counteract cancer cells migration and invasion by acting through inhibition of AKT signaling pathway [53,54]. The overactivation of PI3K/Akt pathway caused an up-regulation of matrix metalloproteinase 2 (MMP-2) and matrix metalloproteinase 9 (MMP-9), which in turn promoted GBM cells migration [55]. Recently, it has been shown that PACAP significantly reduced glioma cells invasion in the rat brain parenchyma [56] by blocking sonic hedgehog-GLI1 (Shh/GLI1) pathway, another signaling cascade implicated in tumor progression [57]. In our previous work, we have demonstrated that PACAP counteracts HIFs expression in GBM cells exposed to DFX-induced hypoxia by acting through PI3K/AKT and MAPK/ERK inhibition [17]. Moreover, the peptide affected the expression levels of VEGF and its receptors in neuroblastoma cells exposed to hypoxia mimetic conditions induced by DFX treatment [40]. Considering the central role of HIF-1α in neo-angiogenesis and EMT event of GBM, we have here evaluated whether PACAP could modulate these events triggered by micro-environmental hypoxia. The ability of PACAP to counteract EMT process has been previously demonstrated in human renal proximal tubule epithelial cells (PTECs) [58]. However, no studies in our knowledge exist regarding the PACAP effect on EMT in cancer, particularly in GBM. In the present paper, we first demonstrated that PACAP and its receptor are expressed either in epithelial or mesenchymal cells of human GBM. Our results have also shown that PACAP decreases the production and release of VEGF in cells exposed to deferoxamine mesylate, a well-known hypoxia mimetic agent. In particular, it reduced tube formation in H5V endothelial cells cultured with GBM conditioned medium. Furthermore, the peptide interfered with EMT in GMB cells by reducing the expression of mesenchymal markers and affecting their migratory capacity. These data provide new insights regarding the multimodal involvement of PACAP in GBM aggressiveness.

## 2. Methods

### 2.1. Human Glioblastoma Samples and Cell Line

Human glioblastoma frozen sections were provided from Anatomic Pathology of “G.F. Ingrassia” Department after patients signed informed consent. The study was approved by the local ethics committee of the Research Center on Motor Activities (CRAM), University of Catania (Protocol Number: CRAM-015-2020, 16/03/2020). Experiments were also carried on human GBM cell line U87MG (wild-type p53) (ATCCC number #HTB-14), obtained from the American Type Culture Collection (ATCC, Rockville, MD, USA). Cells were grown in Dulbecco’s modified Eagle’s medium (DMEM) supplemented with 10% of heat-inactivated fetal bovine serum (FBS), 100 U/mL penicillin and 100-μg/mL streptomycin (Sigma–Aldrich, Steinheim, Germany). Cells were incubated at 37 °C in a humidified atmosphere with 5% CO_2_, as previously described by Maugeri et al. [59].

### 2.2. Treatments

To mimic hypoxic conditions, cells were exposed for 24 h to deferoxamine mesylate (DFX, 100 µM; Sigma–Aldrich), acting through inhibition of the HIF prolyl hydroxylases. Then, 100 nM pituitary adenylate cyclase activating polypeptide-38 (cat no. A1439, Sigma–Aldrich) was added to U87MG cells (50 × 10^4^ cells/dish 75) for 24 h in cells cultured in normoxia or hypoxia mimetic conditions. The treatment with the hypoxia–mimetic iron chelator DFX, as compared to the cell incubation method in hypoxic chamber, offers the advantage to the experimenter to open the culture plate/dish/flask many times without altering the hypoxic condition. However, DFX treatment reproduces a hypoxic event less relevant to that triggered by the use of a hypoxic chamber since it does not directly interfere with the oxygen levels.

### 2.3. ELISA

VEGF-A release in conditioned media were measured using the ELISA sandwich enzymatic method with a specific anti-VEGF-A antibody (RayBio human VEGF-A; Cat. No. ELH-VEGF; RayBiotech Life, Inc., Peachtree Corners, GA, USA) coated on a 96-well plate, according to the manufacturer’s guidelines. Briefly, confluent U87MG cells grown in media supplemented with 1% FBS were treated for 24 h with DFX with or without PACAP. Standards or supernatants from samples were pipetted into the wells containing the immobilized anti-VEGF-A antibody. The wells were then washed before adding biotinylated anti-human VEGF antibody. Following incubation, the unbound biotinylated antibody was washed off, and HRP-conjugated streptavidin was pipetted in each well. After an additional wash step, a 3,3′,5,5′-tetramethylbenzidine (TMB) substrate solution was added to each well, resulting in blue coloration proportional to the amount of bound VEGF. Finally, the stop solution was added, and the colorimetric intensity of the blue substrate, now turned yellow, was measured at 450 nm. The mean absorbance was calculated for each set of duplicate standards, controls and samples, and the average zero standard optical density was subtracted.

### 2.4. Wound Healing Assay

U87MG cells grown to confluence in six-well dishes (5 × 10^4^ cells/well) were scratched with a 200-μL pipette tip, and wound closure was followed. Cells were incubated in 1% serum medium with or without PACAP either in normoxia or under hypoxia mimetic condition. Quantitative assessment of the wound area was performed under an inverted microscope, as previously described [60]. The distance that the advancing cells had moved into the cell-free (wound) area was measured after 24 h. The migration was calculated as the average number of cells observed in three random of high-power wounded fields per well in duplicate wells and expressed in percentage of control (% of control).

### 2.5. Conditioned Medium and Preparation and Tube Formation Assay

Sub-confluent U87MG cell cultures (25 × 10^4^ cells/dish 75) were placed in media supplemented with 1% FBS (conditioned medium 1-CM1; control) or containing 100 nM of PACAP (CM2) or 100 µM DFX (CM3) or DFX plus PACAP (CM4) and incubated at 37 °C for 24 h. After that, the CMs were collected and centrifuged at 2000 rpm for 5 min, and the supernatants were frozen at −80 °C until use. GeltrexTM reduced growth factor basement membrane matrix (Invitrogen, Carlsbad, CA, USA) was thawed at 4 °C overnight before use. GeltrexTM matrix was added to a 24-well plate (95 µL/well) and then incubated at 37 °C for 30 min to allow polymerization. Murine microvascular endothelial cells (H5V) (3 × 10^4^ cells/well) were starved overnight in the growth medium; then, the cells were seeded onto the layer of GeltrexTM matrix and cultured with 200µL of CM1, CM2, CM3 or CM4 at 37 °C for 24 h. Five randomly selected fields of view were captured with a digital camera (Canon, Tokyo, Japan) attached to an inverted microscope (Axio Observer A1; Carl Zeiss, Göttingen, Germany). Tube numbers were calculated as the average tube numbers per fields in duplicate for each well and calculated as percentage of control.

### 2.6. Western Blot Analysis

Proteins were extracted from total cells lysate, as previously described by D’Amico et al. [61]. Briefly, a buffer containing 20 mM Tris (pH 7.4), 2 mM EDTA, 0.5 mM EGTA; 50 mM mercaptoethanol, 0.32 mM sucrose and a protease inhibitor cocktail (Roche Diagnostics, Monza, Italy) was added to U87MG cells adhering to the well using a Teflon-glass homogenizer and then sonicated twice for 20 sec using an ultrasonic probe. Protein concentrations were determined by the Quant-iT Protein Assay Kit (Invitrogen). About 20 µg of protein homogenate was diluted in 2X Laemmli buffer (Invitrogen), heated at 70 °C for 10 min, separated on a Biorad Criterion XT 4–15% Bis-tris gel (Invitrogen) by electrophoresis and then transferred to a nitrocellulose membrane (Invitrogen). Blots were blocked using the Odyssey Blocking Buffer (Li-Cor Biosciences, Lincoln, NE, USA). The transfer was monitored by a pre-stained protein molecular weight marker (BioRad Laboratories, Segrate, MI, Italy). Immunoblot analysis was performed by using specific antibodies: rabbit polyclonal anti-MMP2 (cat. no. ab97779, Abcam, Cambridge, MA, USA); rabbit polyclonal anti-MMP9 antibody (cat. no. ab38898, Abcam); rabbit polyclonal anti-Vimentin antibody (cat. no. ab137321, Abcam); goat polyclonal anti-VEGF (cat. no. sc-1836, Santa Cruz Biotechnology, Dallas, TX, USA), rabbit polyclonal anti-ZO-1 (cat. no. 61-7300, Thermo Fisher, MA, USA) and rabbit polyclonal anti-β-tubulin (cat n.sc-9104, Santa Cruz Biotechnology). The secondary antibodies goat anti-rabbit IRDye 800CW (cat #926-32211; Li-Cor Biosciences), donkey anti-goat IRDye 800CW (cat #926-32214; Li-Cor Biosciences) and goat anti-mouse IRDye 680CW (cat #926-68020D, Li-Cor Biosciences) were used, respectively, at 1:20,000 and 1:30,000. Blots were scanned with an Odyssey Infrared Imaging System (Odyssey), as previously described by Maugeri et al. [62]. Densitometric analyses of Western blot signals were performed by using the ImageJ software 1.8.0_172 (NIH, Bethesda, MD, USA; available at http://rsb.info.nih.gov/ij/index.html; last access date 28 April 2021). Values were normalized to β-tubulin used as loading control.

### 2.7. Immunolocalization

To determine the cellular distribution of PACAP, PAC1R, HIF-1α, vimentin, zonula occludens-1 (ZO-1), MMP-2 and CD44 proteins, immunofluorescence analysis was performed as previously described by D’Amico et al. [63]. Fresh-frozen sections of a surgically resected tumor included in OCT were cut and fixed in 4% paraformaldehyde for 30 min. Then, they were permeabilized with 0.2% Triton X100. To reduce non-specific staining, sections were treated with 1% bovine serum albumin (BSA) in PBS for 1 h and incubated overnight at 4 °C with anti-PACAP (1:100), anti-PAC1R (1:100) and anti-HIF-1α (1:50) and anti-Vimentin (1:50), anti-ZO-1 (1:50) and anti-MMP-2 (1:50) antibodies. Cells were cultured on glass cover slips, fixed in 4 % paraformaldehyde in phosphate-buffered saline (PBS; 15 min at room temperature), permeabilized with 0.2% Triton X-100, blocked with 0.1% BSA in PBS and then probed with anti-Vimentin (1:50), anti-ZO-1 (1:50) and anti-CD44 (1:50) antibodies. Signals were, respectively, revealed with Alexa Fluor 488 goat anti-rabbit or Alexa Fluor 594 goat anti-mouse for 1.5 h at room temperature (shielded from light). DNA was counterstained with 4,6-diamidino-2-phenylindole (DAPI; cat. no 940110, Vector Laboratories, Burlingame, CA, USA). After a series of washes in PBS and double-distilled water, the fixed cells were cover-slipped with Vectashield mounting medium (Vector Laboratories). Immunolocalization was analyzed by confocal laser scanning microscopy (Zeiss LSM700, Zeiss Oberkochen Germany). Green and blue signals were, respectively, detected with laser light at 488 nm/10 mW and 405 nm/5 mW by using the objective “PLANAPOCHROMAT” 63×/1.40 OIL DIC M27. Each scan was individually digitalized by a high sensitivity photomultiplier tube using the following acquisition setup: Gain master: 776; digital offset: −202; digital gain: 1.0. All acquisitions were performed with ZEN-2010 software [64].

### 2.8. Statistical Analysis

Data are represented as mean ± standard error (SEM). One-way analysis of variance (ANOVA) was used to compare differences among groups and statistical significance was assessed by the Tukey–Kramer post hoc test. Colocalization of PACAP and PAC1R with HIF-1α was analyzed using Mander’s overlap coefficient and unpaired t-tests. The level of significance for all statistical tests was set at *p* ≤ 0.05.

## 3. Results

### 3.1. PACAP and PAC1R Expression in GBM Hypoxic Area

**Figure 1:**
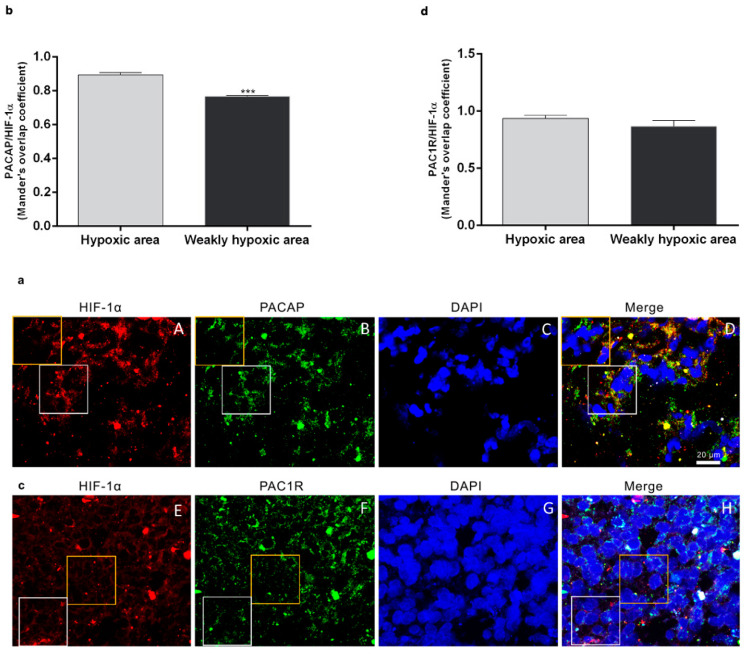
Expression of PACAP and PAC1R in hypoxic area of human GBM sections. (**a**,**c**) Representative photomicrographs showing PACAP (panel B, green), PAC1R (panel F, green), and HIF-1α expression (panels A and E, red) in fresh-frozen sections of a surgically resected GBM. Nuclei were stained with DAPI. The white square indicates a representative hypoxic area showing a higher HIF-1α expression in contrast to a non-hypoxic area limited by a yellow square representing a region with a weak HIF-1α expression. Photomicrographs are representative results of fields taken randomly from each slide and scanned by confocal laser scanning microscopy (CLSM; Zeiss LSM700). (**b**,**d**) PACAP/HIF-1α and PAC1R/HIF-1α expression in hypoxic and weakly hypoxic regions were analyzed by using Mander’s overlap coefficient. Data represent mean ± S.E.M. (*** *p* < 0.001).

To investigate the peptide and receptor expression in the hypoxic area of the tumor, we analyzed the co-localization of PACAP or PAC1R with HIF-1α on human GMB sections by double-immunofluorescence analysis. In Figure 1, we highlighted with a white square the hypoxic area showing higher HIF-1α expression in contrast to a non-hypoxic area limited by a yellow square representing a region with a weak HIF-1α expression. As shown in Figure 1a,c, PACAP and PAC1R are present in the hypoxic region since they co-localize with HIF-1α, the hypoxia biomarker. However, the peptide and receptor were also detected in the non-hypoxic areas showing a weak HIF-1α expression. To quantify PACAP and PAC1R expression in hypoxic or non-hypoxic area, we performed analysis of immunofluorescence signals by using Pearson correlation coefficient and the Mander’s overlap coefficient. As shown in the graph bar of Figure 1b, PACAP/HIF-1α colocalization was markedly reduced in the non-hypoxic GBM area as compared to hypoxic area (*** *p* < 0.001 vs. hypoxic area). No significant difference was detected in PAC1R/HIF-1α colocalization in hypoxic or weak hypoxic GBM area.

**Figure 2:**
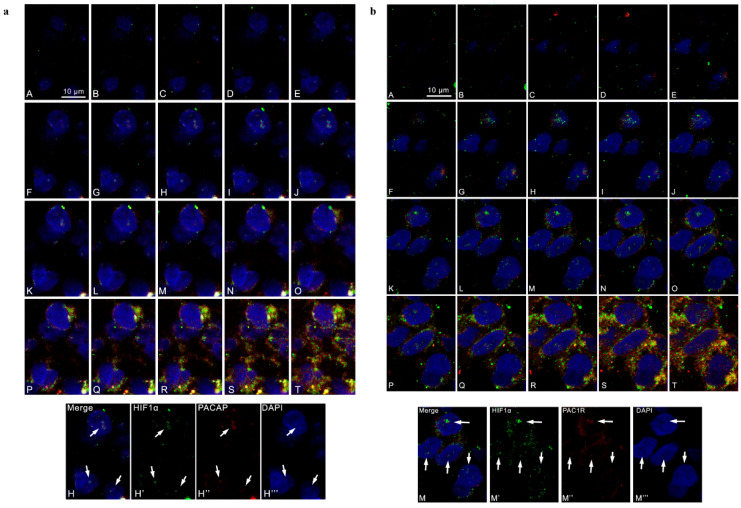
Cellular co-localization of PACAP, PAC1R and HIF-1α in human GBM serial sections. Representative photomicrographs showing the cellular co-localization of HIF-1α and PACAP or PAC1R. (**a**) Green, red and blue correspond to HIF-1α, PACAP and DAPI respectively. H’, H’’ and H’’’ in the panel c show the red, green and blue channels corresponding to a section positioned in the middle part of the z axis. The white arrows indicate the co-localization of HIF-1α and PACAP in the cell nuclei. Scale bar is 10 µm. (**b**) Green, red and blue correspond to HIF1α, PAC1R and DAPI respectively. M’, M’’ and M’’’, in the panel d, show the red, green and blue channels corresponding to a section positioned in the middle part of the z axis. The white arrows indicate the co-localization of HIF1α and PAC1R in the cell nuclei. Scale bar is 10 µm.

To further deepen our analysis, we evaluated the co-localization of HIF-1α and PACAP or PAC1R in cells co-expressing them. The serial section confocal microphotographs reported in Figure 2 allowed us to visualize the sub-cellular co-localization of PA-CAP/PAC1R and HIF-1α in the nucleus (Figure 2, panel a A–H; Figure 2, panel b A–I), perinucleus (Figure 2, panel a I–M; Figure 2, panel b J,K) and cytoplasm (Figure 2, panel a N–T; Figure 2, panel b L–T). As expected, HIF-1α immunoreactivity was detected in all cellular compartments of tumor cell (Figure 2, panel a and b). Interestingly, the double-immunofluorescence analysis showed a cytoplasmic, perinuclear and nuclear co-localization of HIF-1α/PACAP (Figure 2, panel a) or HIF-1α/PAC1R (Figure 2, panel b), confirming a close association among PACAP/PAC1R expression and hypoxic micro-environment. To better visualize the signal of the peptide and the relative receptor, in microphotograph H’’ was highlighted nuclear localization of PACAP, whereas microphotograph M’’ reported nuclear localization of PAC1R and their merging signal with HIF-1α.

### 3.2. PACAP Effect on VEGF Production in GBM Cells Exposed to DFX-Induced Hypoxia

**Figure 3:**
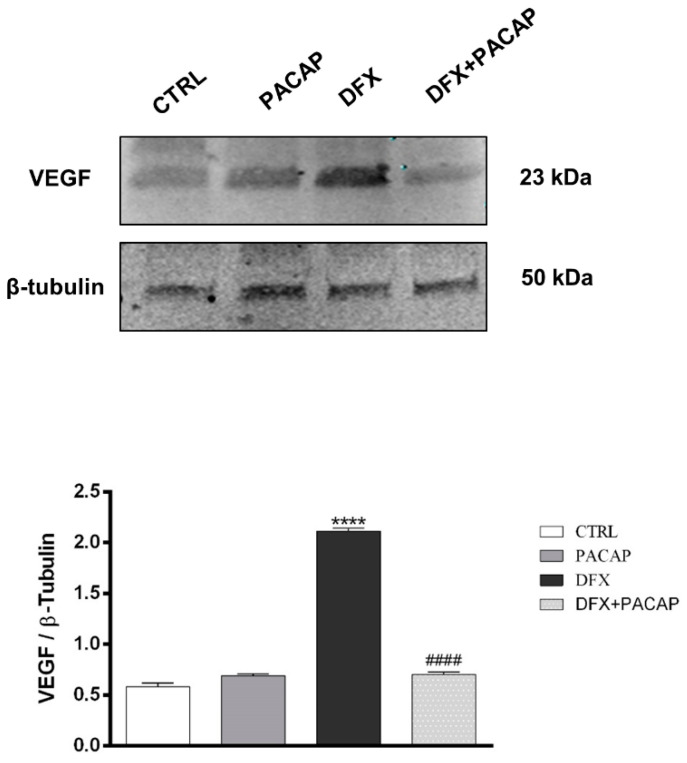
PACAP effect on VEGF expression in human GBM cells exposed to DFX-induced hypoxia. The expression of VEGF was evaluated in U87MG cells treated with vehicle or 100 nM of PACAP or DFX or DFX plus PACAP for 24 h through Western blot analysis. The bar graphs show quantitative analysis of signals obtained by immunoblots resulting from three independent experiments. Relative band densities were quantified by using ImageJ software. Protein levels are expressed as arbitrary units obtained following normalization to β-tubulin, which was used as loading control. Data represent means ± SEM. **** *p* < 0.0001 vs. CTRL; ^####^ *p* < 0.0001 vs. DFX.

To characterize the functional role of PACAP and its receptor on neo-angiogenesis induced by the hypoxic tumor micro-environment, we investigated the effect of the peptide on U87MG cells exposed to DFX. In our previous work, we have demonstrated that PACAP treatment reduced HIF-1α expression in U87MG cells exposed to DFX-induced hypoxia [17]. Since neo-angiogenesis process triggered by low oxygen tension levels is closely associated to VEGF production and release, here we investigated whether PACAP interfered with VEGF expression and secretion in U87MG cells exposed to hypoxic mimetic agent, DFX. As shown in Figure 3, the expression of VEGF was significantly increased in GBM cells following the hypoxic insult (**** *p* < 0.0001 vs. CTRL). The peptide treatment induced significant reduction of intra-cellular VEGF levels in hypoxic condition (^#^ *p* < 0.05 and ^####^ *p* < 0.0001 vs. DFX). Hypoxia mimetic condition also increased secretion of VEGF in supernatant of U87MG cells (**** *p* < 0.0001 vs. CTRL; Table 1), whereas PACAP treatment significantly reduced the growth factor releases in culture medium as demonstrated by ELISA assay (^###^ *p* < 0.001 vs. DFX; Table 1).

In Table 1, we reported VEGF concentration calculated in each conditioned medium derived from U87MG cells cultured with vehicle (CM1), PACAP (CM2), DFX (CM3) and DFX + PACAP (CM4).

### 3.3. PACAP Reduces New Vessels Formation Induced by Conditioned Media from GBM Cell Cultures

**Figure 4:**
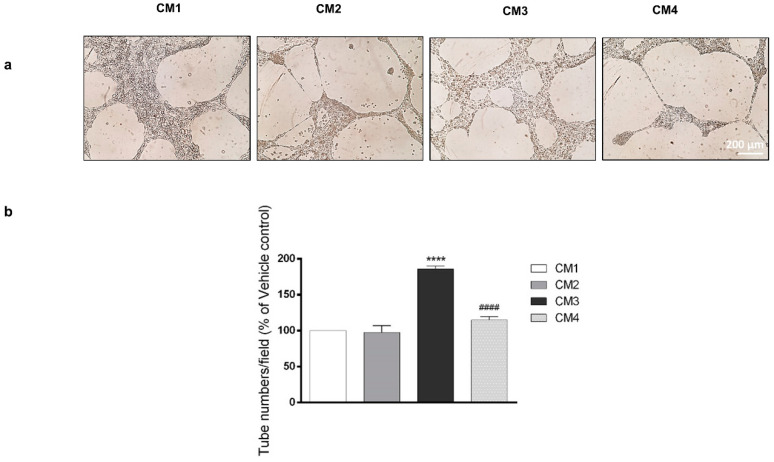
Effect of PACAP on new vessels formation. (**a**) Representative results of tube formation assay. H5V cells were cultured with 200µL of conditioned medium derived from U87MG cells treated with vehicle (CM1), PACAP (CM2), DFX (CM3) or DFX plus PACAP (CM4). The tubes were visualized under a microscope for quantification and calculated as the average tube numbers per field in duplicate for each well. (**b**) In the bar graph, values are expressed as percentage of control (**** *p* < 0.0001 vs. CTRL; ^####^ *p* < 0.0001 vs. DFX).

VEGF secretion in tumor micro-environment leads to neo-angiogenesis. To examine whether PACAP is able to counteract this process, we tested the effect of peptide in a model in vitro of H5V cells forming a network of tube-like structures mimicking neo-vessels formation. Cells were cultured with 200 µL of CM1, CM2, CM3 or CM4 for 24 h. As shown in Figure 4, incubation of H5V cells with CM3, derived from U87MG cells cultured with DFX and containing 7203 pg/mL VEGF, significantly increased the number of tube-like structures as compared to vehicle treated GBM cells (**** *p* < 0.0001 vs. CM1). In contrast, H5V cells cultured with CM4 containing 7013 pg/mL VEGF formed fewer number of tube-like structures as compared to cells grown in CM3 (^####^ *p* < 0.0001 vs. CM3). This result confirmed that the peptide decreases the release of VEGF in the GBM hypoxic micro-environment by reducing new vessels formation.

### 3.4. PACAP Counteracts the Hypoxia Mimetic Condition-Induced EMT in U87MG Cells

**Figure 5:**
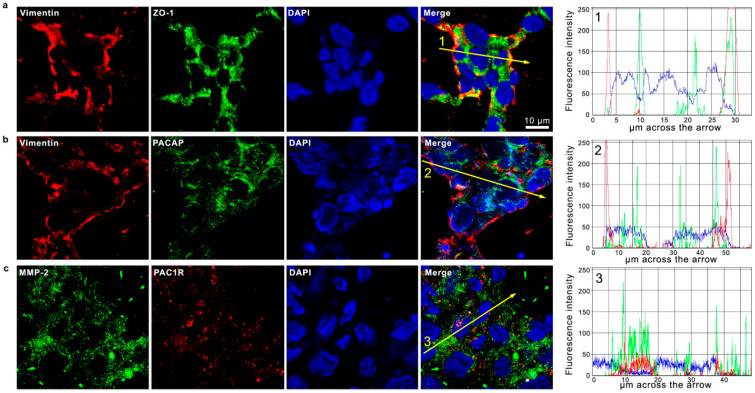
Co-expression of PACAP and PAC1R with EMT-related markers in human GBM sections. Representative photomicrographs showing the cellular co-localization of Vimentin (red) with ZO-1 (green) (**a**); Vimentin (red) with PACAP (green) (**b**); and MMP-2 (green) with PAC1R (red) (**c**). Nuclei were stained with DAPI (blue). Photomicrographs are representative results of fields taken randomly from each slide and scanned by confocal laser scanning microscopy (CLSM; Zeiss LSM700). The graphs indicate the measured fluorescence intensity of the single channels across the arrow, allowing us to visualize the specific peaks of single the proteins or their co-expression detected by overlapped signals. Fluorescence intensity graphs were obtained with ZEN-20210 software.

GBM is a heterogeneous tumor containing different cell types. In particular, its aggressiveness is associated with the EMT process characterized by the conversion of epithelial cancer cells toward the undifferentiated mesenchymal phenotype. To confirm this evidence, we have analyzed the expression of two EMT markers in human GBM sections: vimentin and ZO-1. Vimentin, representing a specific mesenchymal marker, is the major element of the intermediate filament family of proteins and is ubiquitously expressed in normal mesenchymal cells, regulating the maintenance of cell integrity and resistance. Its expression increased in different cancers including GBM [65]. On the contrary, the ZO-1 marker is involved in the assembly of tight and adherent junctions of epithelial cells. In tumor cells, ZO-1 expression decreased following the activation of EMT [66]. As shown in Figure 5a, vimentin was detected in some cells surrounding others expressing ZO-1 (Figure 5a). To characterize the cellular expression of PACAP and PAC1R in human GBM, we have also analyzed their co-localization with vimentin and another mesenchymal marker, MMP-2. As shown in Figure 5b,c, some cells were positive for vimentin and MMP-2, whereas the peptide and its receptor were also diffusely localized in cells immuno-negative to mesenchymal markers. This result suggested that PACAP and PAC1R were expressed in cells with different phenotypes. Previous works have shown that the EMT process is favored by the hypoxic environment [18,19].

**Figure 6:**
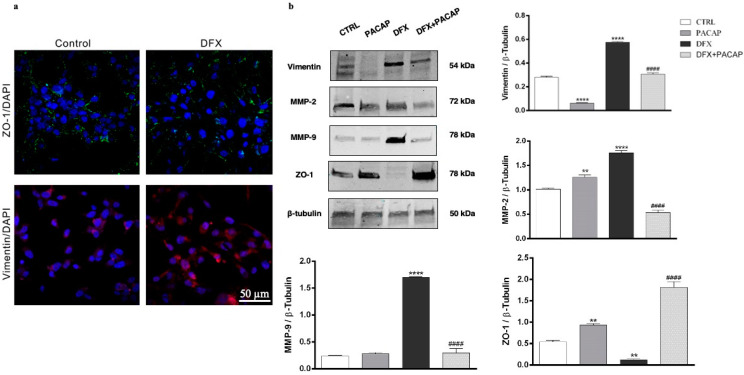
PACAP effect on EMT markers in human GBM cells exposed to DFX-induced hypoxia. (**a**) Immunosignal of ZO-1 (green) and Vimentin (red) expression in U87MG cells cultured in normal oxygen tension levels (control) or in hypoxic condition (DFX) for 24 h. The ZO-1 and Vimentin signals were detected using specific primary antibodies revealed with the Alexa Fluor 488 secondary antibodies (green fluorescence), and Alexa Fluor 594 secondary antibodies (red fluorescence), respectively. Cell nuclei were stained with diamidino-2-phenylindole, DAPI (blue fluorescence). The photomicrographs are representative results taken from different fields in randomly selected slides and scanned by confocal laser scanning microscopy (CLSM). Scale bar (50 µm). (**b**) Representative immunoblots of Vimentin, MMP-2, MMP-9 and ZO-1 protein expression on U87MG cells cultured with PACAP or DFX or DFX plus PACAP or vehicle for 24 h. The bar graphs show quantitative analysis of signals obtained by immunoblots resulting from three independent experiments. Relative band densities were quantified by using ImageJ software. Protein levels are expressed as arbitrary units obtained after normalization to β-tubulin, which was used as loading control. Data represent means ± SEM. ** *p* < 0.01 and **** *p* < 0.0001 vs. CTRL; ^####^ *p* < 0.0001 vs. DFX.

To investigate the functional role played by PACAP on EMT process hypoxia induced, we have studied its effect on U87MG cells exposed to DFX. As shown in Figure 6a, the cell culture included different cell phenotypes, considering that some cells expressed ZO-1, whereas others expressed vimentin. The exposure to DFX induced the EMT event in U87MG cells, as demonstrated by the increased expression of vimentin in contrast to the down-regulation of ZO-1. To investigate whether PACAP was able to counteract the EMT event, we analyzed the expression of some specific markers in GBM cells treated with the peptide and exposed to hypoxia mimetic condition. As shown in Figure 6b, the U87MG cells after DFX insult exhibited higher expression of EMT markers including vimentin, MMP-2 and MMP-9 as compared to control (**** *p* < 0.0001 vs. CTRL). On the contrary, low levels of ZO-1 epithelial marker were detected in DFX group (** *p* < 0.01 vs. CTRL). The treatment with PACAP of cells grown under hypoxia mimetic condition significantly decreased the expression of vimentin, MMP-2 and MMP-9, with a simultaneous increase in ZO-1 expression respect to DFX group (^####^ *p* < 0.0001 vs. DFX) (Figure 6b).

**Figure 7:**
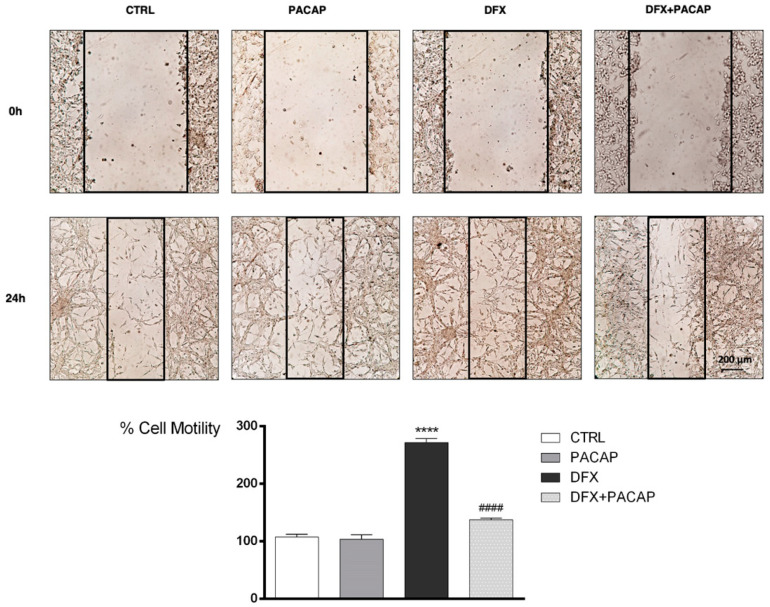
PACAP effect on GBM cells migration following DFX-induced hypoxic insult. Cell mono-layer was scraped by a pipette. The wounded areas were visualized under a microscope for quantification. Migration was calculated as the average number of cells observed in five random wounded fields per well in duplicate wells. In the bar graph, values are expressed as percentage of control. **** *p* < 0.0001 vs. CTRL; ^####^ *p* < 0.0001 vs. DFX.

To evaluate whether PACAP affects GBM cells migration following hypoxic insult, we have performed wound healing assay. As shown in Figure 7, U87MG cells motility drastically increased after exposure to DFX for 24 h as compared to control (**** *p* < 0.0001 vs. CTRL). Instead, fewer cells grown under hypoxia mimetic condition moved toward the wounded area after PACAP-treatment (^####^ *p* < 0.0001 vs. DFX).

**Figure 8:**
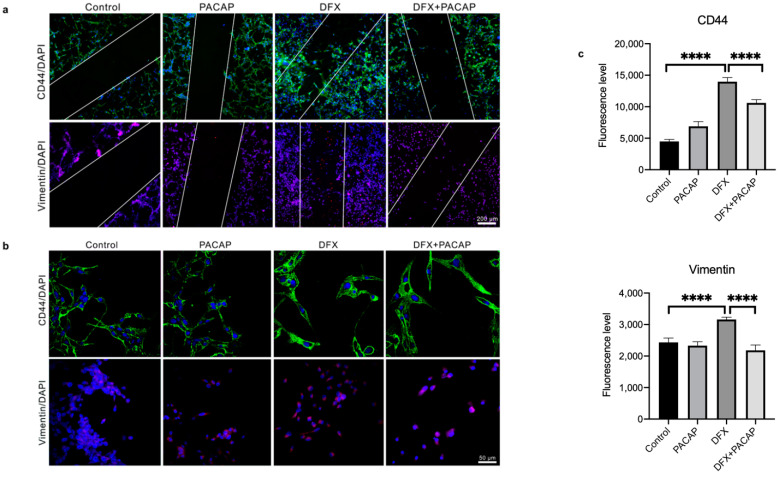
PACAP effect on CD44 and Vimentin expression in migrating GBM cells exposed to hypoxia mimetic conditions. Immunosignal of CD44 and Vimentin protein in U87MG cells cultured with PACAP or DFX or DFX plus PACAP or vehicle for 24 h. (**a**,**b**) The CD44 and Vimentin signals were detected using specific primary antibodies revealed with the Alexa Fluor 488 secondary antibodies (green fluorescence) and Alexa Fluor 594 secondary antibodies (red fluorescence), respectively. Cell nuclei were stained with diamidino-2-phenylindole, DAPI (blue fluorescence). The photomicrographs in panel b represent randomly acquired images from regions surrounding the wound area. Panels a and b show representative results taken from different fields in randomly selected slides and scanned by confocal laser scanning microscopy (CLSM). Scale bars (200 and 50 µm). (**c**) Fluorescence intensities graphs were obtained with the “Profile view” function of ZEN-2011 software; the fluorescence red, green and blue were read for each pixel along the lines, and the graphs reflect, in arbitrary units, the proportion of the pixel intensity in the three wavelengths. Data represent means ± SEM (**** *p* < 0.0001).

To characterize the phenotype of migrant cells along the scratch, we have evaluated the expression of vimentin and CD44 through immunofluorescence analysis. CD44 exerts a key role in GBM invasion [67]. In fact, it has recently demonstrated that GBM cells engage and invade hyaluronic acid-rich matrix through micro-tentacles expressing CD44 receptor [68]. As shown in Figure 8a, an abundant invasion of wound area by cells expressing high levels of CD44 and Vimentin has been observed under hypoxia mimetic condition. Interestingly, PACAP treatment reduced the migratory ability of mesenchymal cells exposed to DFX-induced hypoxia, as demonstrated by the reduced expression of both EMT makers (Figure 8a–c; **** *p* < 0.0001).

## 4. Discussion

GBM, the most common and fatal type of brain tumor, is characterized by high degree of cellular heterogeneity and the strong ability to invade the surrounding tissue. GBM contains areas of intra-tumor necrosis that are typically associated to extensive hypoxic foci. The hypoxic event is principally mediated by HIFs, whose activation induces the transcription of a wide variety of genes involved in several pathways such as glycolysis, angiogenesis, invasion and EMT, all events playing a crucial role in GBM aggressiveness [19]. Our previous study showed that PACAP counteracts the expression of HIFs via PI3K/AKT and MAPK/ERK pathways inhibition [17]. In the present work, we demonstrated that PACAP is mainly expressed in hypoxic areas as compared to weakly hypoxic regions of human GBM (Figure 1a,b). On contrary, PAC1R is diffusely localized in the tumor bulk (Figure 1c,d). In the cell, PACAP/PAC1R showed co-localization with HIF-1α both in the cytoplasm and nucleus (Figure 2). In accord, Yu et al. [69] has previously described the translocation of PAC1R from cytoplasm into the nucleus based on PACAP concentration. At low concentration, the peptide induced the dimerization of PAC1R on perinuclear membrane, whereas at high concentration it promoted translocation of PAC1R dimers into the nucleus triggering different cell signaling. Furthermore, some insults caused up-regulation of PAC1R and its nuclear translocation to protect cells against oxidative stress damage [70]. The presence of PAC1R into the nucleus may be involved in a specific cell response to hypoxia. In accord, PACAP acts as negative regulator of HIF-1α through the inhibition of PI3K/Akt and ERK1/2 signaling pathways, which interfere with HIFs expression [71,72,73]. Although further investigations are needed, the co-localization of HIF-1α with PACAP and PAC1R in the nucleus prompts us to hypothesize that PACAP might inhibit HIF-1α not only through suppression of PI3K/Akt and ERK1/2 pathways but also at activity levels through direct binding with HIF-1α in the nucleus.

It is well-known that HIF-1α plays a key role in the activation of angiogenic cascade by inducing the up-regulation of VEGF [16,74]. To investigate whether PACAP binding to its receptor in the hypoxic area could interfere with neo-angiogenesis, we have analyzed its effect on in vitro model of GBM cells exposed to DFX-induced hypoxia. As shown in Figure 3, VEGF was significantly up-regulated in cells grown under hypoxia mimetic condition as well as its secretion in the culture medium (Table 1). VEGF released in the extracellular micro-environment of GBM actively participates to aberrant angiogenesis. This event is promoted by migration of endothelial cells toward the extracellular matrix, where they adhere to each other by creating a lumen [75]. To confirm the key role exerted by hypoxic micro-environment in the angiogenic process, we cultured H5V endothelial cells in conditioned media from GBM cells exposed to hypoxia mimetic condition (CM3). The predicted prominent pro-angiogenic response has been confirmed by increased number of tubes formation, representing an in vitro model of blood vessels (Figure 4). The peptide treatment decreased the expression levels of VEGF as well as its release (Figure 3 and Table 1). This reduction affected the aberrant angiogenesis as demonstrated by the reduced number of micro-vessels observed when H5V cells were cultured with CM4 (Figure 4). Although VEGF is the primary inducer of angiogenesis released in U87MG CM [76,77], other factors concur to promote the angiogenic response. In accord, Pen et al. [78] showed that transforming growth factors (TGF)-β1 and TGF-β2 released in U87MG-CM induce the migration and invasion of glioma cells as well as the angiogenic event. As highlighted in recent papers, hypoxia through HIF-1α activation participates to increase cell stemness in tumor mass by promoting the EMT and addressing the GBM fate toward malignancy [18,79,80]. GBM is characterized by a high degree of cellular heterogeneity, including epithelial as well as mesenchymal cells. In accord, we have detected in the GBM tissue areas expressing vimentin-positive mesenchymal cells surrounding ZO-1-positive epithelial cells. Interestingly, PACAP and PAC1R are expressed in both cellular phenotypes (Figure 5).

Literature extensively showed that EMT process is promoted by hypoxia [18]. Therefore, we have investigated whether PACAP interferes with this event by analyzing its effect on GBM cells exposed to DFX. U87MG cell culture represents a useful model, in vitro, to study EMT, considering that it contains some cells expressing the epithelial marker ZO1, whereas some others bear the mesenchymal marker Vimentin (Figure 6a). Furthermore, transition toward the malignant phenotype is promoted in cells grown under hypoxia mimetic condition (Figure 6a). In accord, the number of Vimentin immune-positive cells increased after DFX treatment. These data have been confirmed by Western blot analysis. Beside up-regulation of vimentin and down-regulation of ZO-1, GBM cells exposed to hypoxic insult also shown an increased expression of matrix metalloproteinases (MMPs), such as MMP-2 and MMP-9, representing other markers of cellular mesenchymal phenotype (Figure 6b). Up-regulation of these proteins is involved in the degradation of the extracellular matrix and basement membrane, allowing GBM cells to spread to surrounding tissue [81]. It is noteworthy that PACAP interfered with EMT of GMB cells exposed to DFX-induced hypoxia down-regulating Vimentin, MMP-2 and MMP-9 protein levels while, on the other hand, restoring the expression levels of ZO-1 (Figure 6b). As shown in Figure 6b, DFX plus PACAP-treated cells displayed overexpression of ZO-1 as well as down-regulation of MMP-2 more intense compared to control group levels. In our previous paper, we observed a similar result related to ZO-1 expression in retinal pigmented epithelial cells exposed to high glucose and DFX [33]. Although the mechanisms involved are still unknown, we cannot rule out that PACAP could interfere with the expression of these proteins under hypoxia.

Hypoxia increased cell invasive capacity as shown by wound healing assay. In fact, DFX treatment for 24 h increased the number of migrating cells in the wound area as compared to control group. PACAP treatment reduced cells migration along the lesioned region in respect to DFX treated group (Figure 7). Moreover, most migrating cells exposed to hypoxia mimetic condition showed a mesenchymal phenotype, as confirmed by their immune-positive signal for Vimentin and CD44, detected through immunofluorescences analysis (Figure 8). The key role of CD44 in regulation of EMT has been previously demonstrated [82]. It is a non-kinase transmembrane glycoprotein that acts as a receptor for hyaluronic acid, a major element of the extracellular matrix. Considering that the brain contains abundant levels of hyaluronic acid, CD44 could play important role in glioma cells’ migration. In accord, CD44, highly expressed in 55.55% of GBM, induces acquisition of more malignant abilities, and patients with higher levels of this glycoprotein exhibit a shorter survival time [83]. Here, we confirmed the ability of PACAP to prevent cells’ invasiveness by reducing their transition toward the more malignant mesenchymal phenotype. In fact, the peptide treatment reduced the number of Vimentin and CD44 immuno-positive migrating cells (Figure 8). In vitro studies have some limitations since cells are not exposed to the complex biological condition characterizing the tissue in vivo. Therefore, we are planning to study the effects of this neuropeptide on GBM progression in vivo animal models. Moreover, in a future study, we are going to investigate the role of this peptide in GBM cells with different genotype. We have also carried out a preliminary study on T98G cells (Figure S1). Similarly to the results obtained in the U87MG cells, we found that PACAP affects VEGF expression and release in T98G cells exposed to DFX-induced hypoxia. Moreover, we detected a reduced number of tubes formed by H5V cells grown in conditioned medium derived from T98G cells. These cells bear a p53 mutation and a high MGMT activity and, as demonstrated in a recent paper, these genomic characteristics make them more resistance to Temozolomide (TMZ) treatment as compared to other GBM cells, such as A172 cells [84]. This investigation might underline the efficacy of the peptide in a combinatory therapy with TMZ for GBM treatment with a specific genotype. Although in the last years progress has been made in the treatment of GBM, patient survival is still very poor. The aggressive phenotype, the invasiveness and the resistance to chemotherapy and radiotherapy of GBM have been linked to its histopathological heterogeneity and multimodal events triggered by tumoral hypoxic micro-environment. The complex regulating mechanism carried out by PACAP might open a new avenue in the therapeutic approach to this fatal malignancy.

## Figures and Tables

**Table 1:** VEGF content in conditioned medium from U87MG cells. VEGF levels were detected in supernatants and expressed in pg/mL. Data resulting from three independent experiments are represented as means ± SEM. **** *p* < 0.0001 vs. CTRL; ^###^ *p* < 0.001 vs. DFX.

U87MG Cell Line-Derived Conditioned Media	CM1 (Vehicle) Mean ± SEM	CM2 (PACAP) Mean ± SEM	CM3 (DFX) Mean ± SEM	CM4 (DFX + PACAP) Mean ± SEM
VEGF (pg/mL)	6584 ± 9.85	6041 ± 5.77 ****	7203 ± 8.54 ****	7013 ± 11.55 ^###^

## Data Availability

All data are available within the manuscript and upon request to the corresponding author.

## Supplementary Materials

The following are available online at https://www.mdpi.com/article/10.3390/biomedicines9080965/s1, Figure S1: The effect of PACAP on angiogenic response in T98G cells exposed to DFX-induced hypoxia.
