# Supplementary figures and images for: Effect of PACAP on Hypoxia-Induced Angiogenesis and Epithelial–Mesenchymal Transition in Glioblastoma
Source: Biomedicines. 2021 Aug 5;9(8):965. doi: 10.3390/biomedicines9080965 (PMC8392618; doi:10.3390/biomedicines9080965)

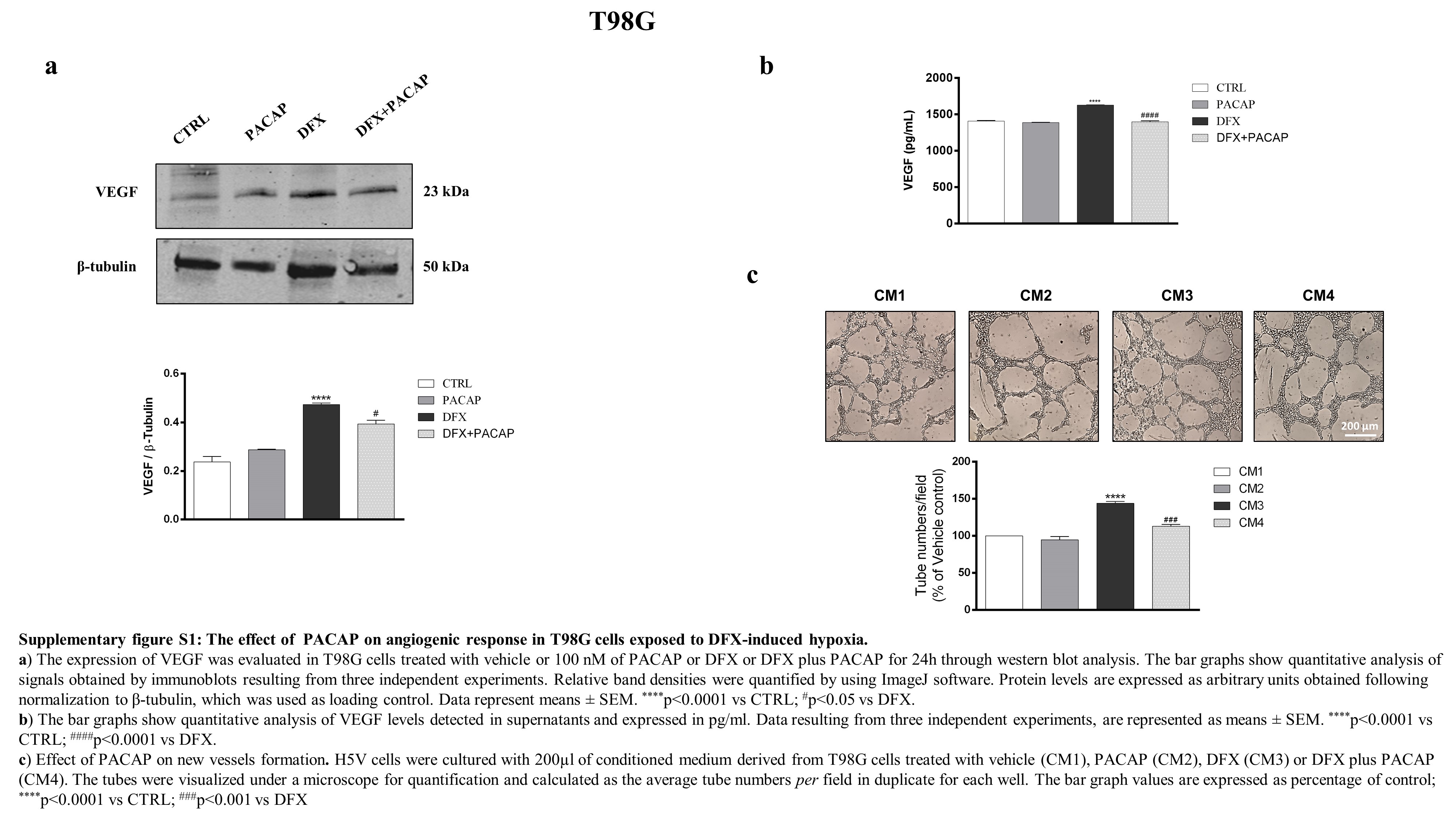

Supplement: Supplementary file 1 [file biomedicines-09-00965-s001.zip › biomedicines-1255564-supplementary.jpg]
